# New Evidence for Strategic Differences between Static and Dynamic Search Tasks: An Individual Observer Analysis of Eye Movements

**DOI:** 10.3389/fpsyg.2013.00008

**Published:** 2013-01-29

**Authors:** Christopher A. Dickinson, Gregory J. Zelinsky

**Affiliations:** ^1^Department of Psychology, Appalachian State UniversityBoone, NC, USA; ^2^Department of Psychology, Stony Brook UniversityStony Brook, NY, USA

**Keywords:** visual search, eye movements, search strategies, memory in search

## Abstract

Two experiments are reported that further explore the processes underlying dynamic search. In Experiment 1, observers’ oculomotor behavior was monitored while they searched for a randomly oriented T among oriented L distractors under static and dynamic viewing conditions. Despite similar search slopes, eye movements were less frequent and more spatially constrained under dynamic viewing relative to static, with misses also increasing more with target eccentricity in the dynamic condition. These patterns suggest that dynamic search involves a form of sit-and-wait strategy in which search is restricted to a small group of items surrounding fixation. To evaluate this interpretation, we developed a computational model of a sit-and-wait process hypothesized to underlie dynamic search. In Experiment 2 we tested this model by varying fixation position in the display and found that display positions optimized for a sit-and-wait strategy resulted in higher *d*′ values relative to a less optimal location. We conclude that different strategies, and therefore underlying processes, are used to search static and dynamic displays.

## Introduction

We often search for things in static displays, situations in which the elements of the scene through which we are searching remain in the same locations over time. The assumption of static search has even pervaded search theory, where it is commonly believed that there is a memory for distractors that have been inspected and rejected, and that this memory is used to improve search efficiency (Treisman and Gelade, 1980; Koch and Ullman, 1985; Treisman, 1988; Treisman and Sato, 1990; Wolfe, 1994; Zelinsky, 2008). A frequently suggested mechanism for distractor memory is the application of inhibition to an item’s location following that item’s rejection during search (Klein, 1988; Klein and MacInnes, 1999; Takeda and Yagi, 2000), an idea related to the process of inhibition of return (Posner and Cohen, 1984). As noted by Alvarez et al. (2007), however, object motion and changes in the observer’s viewpoint can result in objects changing location abruptly and unexpectedly during search, an observation that casts doubt on the theoretical assumption of a memory for rejected distractors.

The hypothesis that distractor memory is used during search was challenged most directly by Horowitz and Wolfe (1998) (see also Smith and Henderson, 2011), who had observers search displays in which items either retained their locations throughout a trial (the static condition) or were randomly relocated every 111 ms (herein referred to as the “dynamic condition”). A memory-driven search process predicts that in the dynamic condition, search should be less efficient than in the static condition because any visual markers (e.g., Watson and Humphreys, 1997) set to item locations in frame *n* of a dynamic display would not be valid in frame *n* + 1, leading to the resampling of items and less efficient search. However, Horowitz and Wolfe (1998) found that search efficiency was comparable in the two display conditions and concluded that memory was not used during search of static displays, despite its availability.

This bold claim was itself challenged almost immediately by Kristjansson (2000) and Shore and Klein (2000). They raised several issues with the Horowitz and Wolfe (1998) paradigm and results, noting that onsets during dynamic search trials, caused by an object appearing at a previously empty location, could have influenced attentional allocation, and that static and dynamic search slopes diverged for set sizes beyond the range tested by Horowitz and Wolfe (1998). Horowitz and Wolfe (2003) addressed these concerns by including a condition that used Kristjansson’s (2000) dynamic search paradigm, testing larger set sizes, and slowing dynamic display changes from 10 to 2 Hz, and again found similar search efficiency for search of static and dynamic displays, consistent with their earlier findings.

The experiments conducted by Horowitz and Wolfe (1998) sparked substantial interest in the empirical study of the memory in search hypothesis (for studies using RT measures, see Gibson et al., 2000; Kristjansson, 2000; Müller and von Mühlenen, 2000; Shore and Klein, 2000; Oh and Kim, 2004; cf. Wolfe et al., 2000; Horowitz and Wolfe, 2001, 2003; Woodman et al., 2001; Horowitz, 2006; Horowitz et al., 2009; for studies using oculomotor measures, see Gilchrist and Harvey, 2000; Peterson et al., 2001; Aks et al., 2002; Boot et al., 2004; Dickinson and Zelinsky, 2005, 2007; Beck et al., 2006). The present study does not address the ongoing debate over whether and how memory is used during search, it focuses instead on the unexpected and compelling finding that sparked this debate: that the search of a display in which items were randomly relocated at a very high frequency could proceed very efficiently. What mechanism underlies efficient search under these conditions? Since Horowitz and Wolfe’s (1998) initial investigation, several other studies have used dynamic displays in an attempt to answer this question. For example, Alvarez et al. (2007) showed that search processes can exploit spatiotemporal continuity and continuity in the global configuration of display items across dynamic display changes. Their data, however, did not reveal how observers efficiently searched the dynamic displays in Horowitz and Wolfe’s (1998) experiment when these two sources of continuity were not present. In a similar vein, Hulleman (2009, 2010) also compared search of static vs. moving objects, but the fact that objects moved in linear trajectories introduced confounds with predictability that complicate interpretations.

One prominent explanation for the Horowitz and Wolfe (1998) dynamic search data is that observers simply maintained gaze at a given display location and monitored it for the target – deemed a “sit-and-wait strategy.” This was suggested initially by Horowitz and Wolfe (1998) but was rejected because monitoring a single item location would have produced far more errors than they observed. von Mühlenen et al. (2003) tested the hypothesis that a sit-and-wait strategy would not result in efficient search of dynamic displays by using an “aperture” condition to force participants to restrict their search to only one quadrant of the dynamic display. They found that search was as efficient, and nearly as accurate, in the aperture condition as when the full display was visible, concluding that a sit-and-wait strategy could be an effective means of searching dynamic displays. Wang et al. (2010) reached a similar conclusion based on combining search of static and dynamic displays with a probe-detection task.

Following von Mühlenen et al. (2003), Geyer et al. (2007) monitored observers’ eye movements during a near replication of Horowitz and Wolfe’s (1998) (Experiment 2) static and dynamic search tasks. The RT and accuracy data were very similar to that found by Horowitz and Wolfe (1998) (Experiment 2): significantly faster search of static displays; no statistical difference in search efficiency (as indexed by the search slopes) between static and dynamic displays when a target was present; significantly more efficient search for dynamic displays when a target was absent; and significantly fewer misses for static displays. They also found different patterns of oculomotor behavior during the search of static and dynamic displays that supported the hypothesis that different strategies underlie these two tasks. For search of static displays, they found a pattern consistent with an active search strategy: fixation number, but not fixation duration, increased with set size, with more fixations when the target was absent. In contrast, for search of dynamic displays, they found a pattern consistent with a passive search strategy: fixation duration, but not fixation number, increased with set size. Taken together, the results of these two studies support the use of fundamentally different strategies for searching dynamic displays as opposed to static displays. Converging evidence for the effectiveness of a passive sit-and-wait search strategy comes from the results of several studies showing that in displays of moving objects, changes were detected more accurately when participants used a passive search strategy than when they used an active search strategy (Boot et al., 2006; Becic et al., 2007, 2008).

The current experiments further explore the hypothesis that a sit-and-wait strategy underlies the search of dynamic displays, focusing on the relationship between eye position and attentional allocation during this search task. In Experiment 1, we examined the role of target eccentricity in dynamic search by using the Geyer et al. (2007) paradigm but presenting targets at one of two display eccentricities with equal probability. Based on the oculomotor and accuracy behavior observed in this experiment, we developed a model of a sit-and-wait process hypothesized to underlie the search of dynamic displays. In Experiment 2, we tested two of the model’s predictions by having participants search dynamic displays while maintaining fixation either at a location predicted by the model to yield suboptimal search accuracy (the display’s center) or at a location predicted by the model to yield optimal search accuracy (midway between the two sets of target eccentricities). We elected to adopt the Geyer et al. (2007) paradigm rather than the Horowitz and Wolfe (2003) paradigm because in the study by Horowitz and Wolfe it is possible that participants could have made multiple fixations within each 500-ms dynamic display – something that would not have been possible in either the Geyer et al. (2007) experiment or in the Horowitz and Wolfe (1998) experiments.

Our methodology differs from Geyer et al. (2007) in two important respects. First, we adopted an individual observer analysis of oculomotor behavior. This allowed us to document the spatial and temporal distributions of fixations during both search tasks and to determine how these characteristics corresponded to their response time and error data. We therefore gain from such an analysis a direct look at the moment-to-moment processing during dynamic search, and a better understanding of the attentional limitations underlying a sit-and-wait search strategy. Notably, Geyer et al. (2007) did not document the spatial distribution of fixations in either of their search tasks (other than noting mean saccade-amplitude differences). Second, we manipulated target eccentricity. We did this for two reasons. First, effects of increasing target eccentricity are well known in the case of static search, with RTs and errors both increasing with target eccentricity in a conjunction search task (Carrasco et al., 1995; Scialfa and Joffe, 1998; Wolfe et al., 1998). However, errors related to eccentricity tend also to decline if observers reposition their gaze before the search judgment (Scialfa and Joffe, 1998). To date, these effects have not been documented for dynamic search. Knowing how target eccentricity influences the search of dynamic displays could provide insight into the processes underlying dynamic search, including the relationship between attentional deployment and eye position. Second, an unintended bias in display characteristics may have contributed to search efficiency in several previous investigations of dynamic search; targets were more likely to appear at eccentric locations than more central ones because there were more eccentric display locations than central locations (Horowitz and Wolfe, 1998; von Mühlenen et al., 2003; Geyer et al., 2007; Wang et al., 2010). Controlling target eccentricity might reveal limits in the efficiency of dynamic search that observers in these studies may have been able to overcome because of these display biases.

## Experiment 1

Horowitz and Wolfe (1998, 2003) interpreted their finding of similar search slopes in their static and dynamic conditions as evidence for these two search tasks tapping into the same underlying search process. However, search set size effects derived from manual RTs are notoriously ambiguous with regard to the processes used in their generation (Palmer, 1996; Wolfe, 1998; Zelinsky, 1999), and it has long been known that similar set size effects can be obtained from dramatically different search processes (e.g., serial vs. limited-capacity parallel processes; Townsend, 1976). Following Zelinsky and Sheinberg’s (1995, 1997) use of an oculomotor analysis to tease apart serial and parallel search processes, can a similar oculomotor characterization of static and dynamic search help to clarify whether these tasks are served by the same or different processes?

Considerable work has documented the movement of gaze in static search tasks (see Rayner, 1978, 1998, 2009; Findlay and Gilchrist, 2003; Zelinsky, 2008, for reviews), with one clear finding being that observers, if not prevented from doing so, will soon cover a display with their gaze as they search for a difficult-to-locate target (Gilchrist and Harvey, 2000; Peterson et al., 2001; Aks et al., 2002; Dickinson and Zelinsky, 2005, 2007). However, there has been only one published report of gaze direction during a dynamic search task (Geyer et al., 2007).

Our premise is that the enlistment of different search processes in these two display conditions might be evidenced by different patterns of eye movements in individual observers. One pattern might involve the active repositioning of gaze during search, presumably accompanied by the active movement of attention to different display positions. Indicating such an active search strategy would be an increase in the number of fixations with both set size and target eccentricity, but little or no change in fixation duration or accuracy. A contrasting pattern, one indicating a sit-and-wait search strategy, would show fewer fixations that were spatially constrained to the sit-and-wait location regardless of set size and target eccentricity. This is the general pattern of oculomotor data reported by Geyer et al. (2007). Given that attention might be used to sample multiple locations surrounding the locus of gaze (Motter and Holsapple, 2007; Motter and Simoni, 2007), a sit-and-wait strategy might also be expected to involve the monitoring of multiple display locations (for a conceptually related idea, see also Courtney and Chan, 1986; Chan and Chiu, 2009). However, the locations monitored as part of such a *multi-location sit-and-wait strategy* would be those nearest the current gaze location (e.g., Wolfe et al., 1998), meaning that observers sitting and waiting near the display’s center may frequently miss targets appearing at eccentric display positions. Finding evidence for these different patterns of gaze behavior and misses in static and dynamic search tasks would suggest that these tasks are served by different search processes.

### Materials and methods

#### Participants

One of the authors and three experimentally naïve observers from Stony Brook University’s undergraduate and graduate communities participated in the experiment. The naïve observers were paid $10/h for their participation and all observers had either normal or corrected-to-normal vision.

#### Apparatus and stimuli

**Figure 1:**
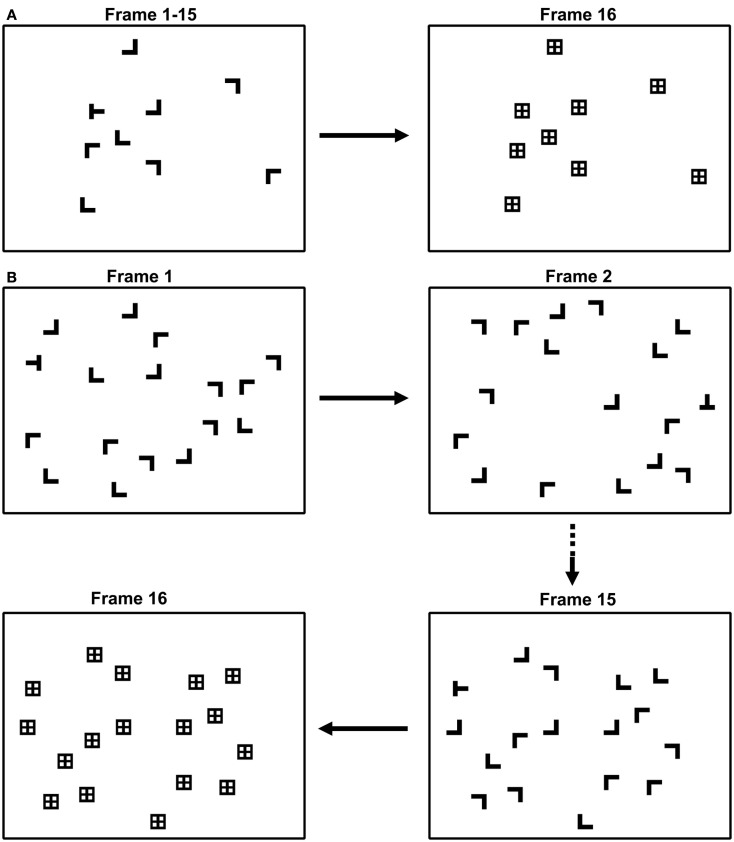
**Samples of (A) static and (B) dynamic search trials**. Static trials depicted a single configuration of search items. Dynamic trials consisted of 15 135 ms displays, each depicting a randomly relocated configuration of search items. Individual items were white and presented on a dark background.

The target was a T, the distractors were Ls, and all items could appear rotated 0°, 90°, 180°, or 270°. Objects were located on three concentric circles whose radii subtended 1.5°, 3.1°, and 4.6°, and a fourth broken circle banding the left and right sides of the display whose radius subtended 6.2°. Item locations at each eccentricity were equally spaced around the respective imaginary circles; across eccentricities, locations were not aligned. Each eccentricity contained 4, 10, 15, and 10 object locations, respectively. Distractor locations were randomly selected from among these 39 display locations; the target appeared only at the second (3.1°) and fourth (6.2°) eccentricities, appearing at each on 50% of the target-present trials. Observers were therefore not biased by target contingencies into shifting gaze to a particular display eccentricity. Individual stimuli subtended 0.75° × 0.75° and were composed of lines 0.19° in width. The minimum center-to-center distance between objects was 1.55°, and all objects were white, presented on a black background. Mask items replaced each search item after a time-terminated display interval. Individual masks consisted of the superimposed target and distractor line segments (i.e., a “+” sign in a square) and subtended 0.75° × 0.75°. Figure 1 shows samples of static and dynamic displays. Eye movements were monitored using a Fourward Technologies Generation VI dual Purkinje image (DPI) eye tracker. This eye tracker has a spatial resolution of better than 0.01° at the experimental viewing distance of 137.5 cm. Eye position was sampled at 1000 Hz using a Prairie Digital analog-to-digital converter.

#### Procedure and design

Participants were instructed to search for the letter T, regardless of its orientation, and to respond as quickly and accurately as possible. A target-present response was indicated by left-clicking a two-button mouse; target absence was indicated by a right-click.

Each trial began with the presentation of a centrally located fixation cross. Observers initiated a trial by pressing a mouse button, which removed the cross and caused the search display to appear. Static trials consisted of a single search display depicting a single configuration of items (item configurations varied from trial to trial). Dynamic trials consisted of 15 sequentially presented displays (configurations varied both within and between trials)^1^. Items were positioned randomly in each display with the constraints that (1) the target appeared at a single eccentricity on a given trial, and (2) a target had to appear in each of the 10 allowable target locations before these locations could be repeated. Each display frame in the dynamic condition was visible for 135 ms. All trials were terminated by a spatial mask after 2,025 ms. A mask item replaced each search item after the time-terminated display interval elapsed. The mask was presented for at least 500 ms, with the actual duration depending on the observer’s response. If the observer made a search judgment prior to the fixed 2,025 ms search exposure, the mask would appear for 500 ms and then be replaced by a display providing RT and accuracy feedback (the words “CORRECT” or “INCORRECT”). If the observer failed to make a judgment during the 2,025 ms exposure, the mask would remain visible until a response was indicated, followed immediately by the feedback. Each observer’s head was stabilized by a dental impression bite bar and two forehead restraints throughout the experiment. Other than instructions to fixate the central cross between trials, observer eye movements were not constrained during the experiment.

Observers participated in 640 trials over two 1-h sessions, conducted on separate days. These 640 trials were evenly divided into 2 set sizes (9 or 17 items), 2 target conditions (present or absent), 2 search conditions (static or dynamic), and 2 target eccentricities (3.1° or 6.2°), leaving 40 trials per target-present cell and 80 trials per target-absent cell.

### Results and discussion

#### Manual button press data

**Figure 2:**
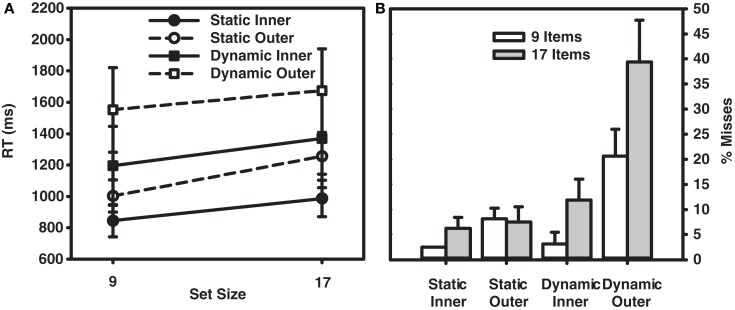
**(A)** RT × Set Size functions for correct responses in the target-present trials from Experiment 1. **(B)** Percent misses as a function of set size and eccentricity (inner and outer) for participants in Experiment 1. Error bars indicate the standard error of the mean.

Prior to examining the data, approximately 4% of the trials were excluded because of manual RTs falling above or below two SDs from the cell mean. Figure 2A shows the target-present RT data, and Figure 2B shows the corresponding miss rates. The average RT and error data from target-absent trials are provided in Table 1, and the button press data for individual observers are provided in Table 2.

**Table 1:** **Mean reaction times and error rates for target-absent trials in Experiments 1 and 2**.

	Experiment 1			
Condition	Static		Dynamic	
Set size	9	17	9	17
RT (ms)	1257	1721	1867	1911
Errors (%)	0.6	3.1	23.1	20.0

**Experiment 2**				
**Condition**	**Central dynamic**		**Eccentric dynamic**	

Set Size	9	17	9	17
RT (ms)	1623	1678	1941	1961
Errors (%)	18.1	25.3	11.1	9.5

*Reaction times were calculated for correct responses only*.

**Table 2:** **Manual reaction times and error rates for individual observers**.

	Target-present								Target-absent			
Condition	Static				Dynamic				Static		Dynamic	
Set size	9		17		9		17		9	17	9	17
Eccentricity	Inner	Outer	Inner	Outer	Inner	Outer	Inner	Outer				
RT (ms)												
G.Z.	959	1006	1056	1252	1138	1331	1193	1546	1450	1799	1831	1842
S.L.	1009	1210	1244	1552	1883	2383	2213	2383	1466	2063	2644	2731
R.C.	712	798	820	1110	975	1141	1173	1321	1034	1454	1594	1596
S.M.	566	827	700	1036	650	1274	716	1356	1077	1570	1398	1474
Mean	812	960	955	1238	1162	1532	1324	1652	1257	1722	1867	1911
Errors (%)												
G.Z.	2.5	12.5	12.5	12.5	10.0	22.5	20.0	47.5	0	2.5	26.3	17.5
S.L.	2.5	10.0	5.0	5.0	2.5	27.5	17.5	42.5	0	0	22.5	18.8
R.C.	2.5	2.5	5.0	0	0	5.0	2.5	15.0	0	0	20.0	27.5
S.M.	2.5	7.5	2.5	12.5	0	27.5	7.5	52.5	2.5	10.0	23.8	16.3
Mean	2.5	8.1	6.3	7.5	3.1	20.6	11.9	39.4	0.6	3.1	23.2	20.0

*Reaction times were calculated for correct responses only*.

Turning first to the static condition, the data reveal a fairly unremarkable pattern of results. Target-present search averaged 26 ms/item, nearly half the 57 ms/item rate found in the target-absent data. Consistent with the literature, search efficiency in the static condition also interacted with target eccentricity. Search proceeded at a rate of 18 ms/item for inner-eccentricity targets, and 35 ms/item for outer-eccentricity targets. The error data accompanying these RTs were equally unremarkable. Misses occurred on less than 10% of the trials and did not vary appreciably with set size or target eccentricity. False alarms were rare, occurring on less than 4% of the trials.

Data from the dynamic search condition were less straightforward and provided mixed support for the patterns reported in Horowitz and Wolfe (1998). Consistent with this earlier study, target-present search of the dynamic displays, when averaged across eccentricity, proceeded at a rate of 18 ms/item – clearly no less efficient than search in the static condition. Also consistent with Horowitz and Wolfe (1998) is the relatively shallow slope (5 ms/item) observed in our target-absent search data^2^. A breakdown of the data by eccentricity, however, reveals two patterns not reported in Horowitz and Wolfe (1998). First, the eccentricity effect that characterized RTs in the static search condition is now gone. Slopes for inner-eccentricity targets averaged 20 ms/item; outer-eccentricity slopes averaged 16 ms/item. Informing this similarity in slope is an analysis of misses by eccentricity. The miss rates for inner-eccentricity targets were 3 and 12% in the 9- and 17-item displays, respectively – in line with the miss rates reported in Horowitz and Wolfe (1998). A very different picture emerged for outer-eccentricity targets. Outer-eccentricity targets were missed on 21% of the 9-item trials and almost 40% of the 17-item trials. From Table 2 it is clear that observers S.L., S.M., and G.Z. were unable to perform the search task when the display contained 17 items and the target appeared at the outer eccentricity, with only the data from R.C. preventing judgments from being at chance.

If observers engaged in a dynamic search task elected to keep their gaze at the display’s center and covertly process the items surrounding fixation, as predicted by a multi-location sit-and-wait search strategy, then outer-eccentricity targets might never be inspected regardless of the number of dynamic frames. The consequence of such a strategy would be a higher miss rate in the outer-eccentricity dynamic condition – the exact pattern observed in the data. Likewise, if observers in the static display condition adopted a more active search strategy, then even outer-eccentricity targets would eventually be inspected. The consequence of such an active search strategy, assuming that covert search originates from the center of gaze and proceeds outward (Wolfe et al., 1998), would be an attenuated effect of eccentricity on misses but a larger effect of eccentricity on RTs – again, the exact pattern observed in our data.

Although the selective use of different strategies to search static and dynamic displays would seem consistent with the current data, one might argue that a sit-and-wait strategy does not well describe data from the Horowitz and Wolfe (1998) study. Error rates in their study, at their very highest, reached only 8%; well below the 39% error rate obtained in our 17-item outer-eccentricity condition. Even when averaged over eccentricity, misses in our dynamic condition were 19%, much higher than those reported by Horowitz and Wolfe (1998) and Geyer et al. (2007), who reported miss rates of approximately 10%.

Can the current evidence in support of a sit-and-wait strategy be reconciled with the relatively low miss rates reported by Horowitz and Wolfe (1998)? Although these authors did not explicitly manipulate target eccentricity, the display configurations used in their dynamic search condition may nevertheless have encouraged observers to adapt a sit-and-wait strategy to deal with eccentric targets. In Horowitz and Wolfe’s (1998) Experiment 2, targets were equally likely to appear in any of 40 display locations. However, because there were more peripheral locations than central ones, the probability of a target in the dynamic condition appearing at the outer two eccentricities (0.7) was higher than the probability of a target appearing at the two inner eccentricities (0.3). Likewise, because von Mühlenen et al. (2003) and Geyer et al. (2007) modeled their displays to match those used by Horowitz and Wolfe (1998), these studies would have been subject to similar target-distribution biases. If observers learned this contingency, they might have adopted the practice of shifting gaze away from the display’s center and sitting and waiting for the target at a more favorable display location – one that positioned gaze nearer to one of the outer-eccentricity target rings. Rather than discouraging the use of a sit-and-wait strategy by having the majority of targets appear at eccentric display locations, these previous studies might therefore have inadvertently biased observers to make an eye movement and thereby create the conditions under which such a strategy could be used to search a dynamic display with a high degree of accuracy. The following analyses explore this relationship between sit-and-wait strategies and gaze position and attempt to discern differences between static and dynamic search behavior in oculomotor variables.

#### Eye movement data

To better assess the possibility that static and dynamic search differences reflect different underlying search processes, we analyzed the number of fixations and the fixation-duration data from individual observers. We expect that a sit-and-wait strategy will be characterized by few, if any, changes in gaze location, and that increases in set size and target eccentricity will instead result in longer fixation durations. The hypothesized effect of set size follows from the assumption that a sit-and-wait strategy involves the monitoring and accumulation of information over a limited display region surrounding current fixation, and that more items will fall within this region as set size increases. The hypothesized effect of eccentricity follows from the assumption that items nearer fixation are processed preferentially relative to more eccentric items, similar to the central attentional bias concept advanced by Wolfe et al. (1998). The logical alternative to a sit-and-wait strategy is what we will term an active acquisition strategy. Rather than sitting and waiting for a target to appear in one of the monitored display locations, a search process may actively seek out the target by inspecting individual items or small fixed-size groups of items. Here we assume that the display region from which information is acquired changes throughout search (Treisman, 1988; Wolfe, 1994), and that some or all of these changes are indicated by changes in gaze position (Zelinsky et al., 1997; Findlay and Gilchrist, 2001). Such a strategy would be characterized in oculomotor variables by an increase in the number of fixations with set size and eccentricity, and relatively constant fixation durations.

**Figure 3:**
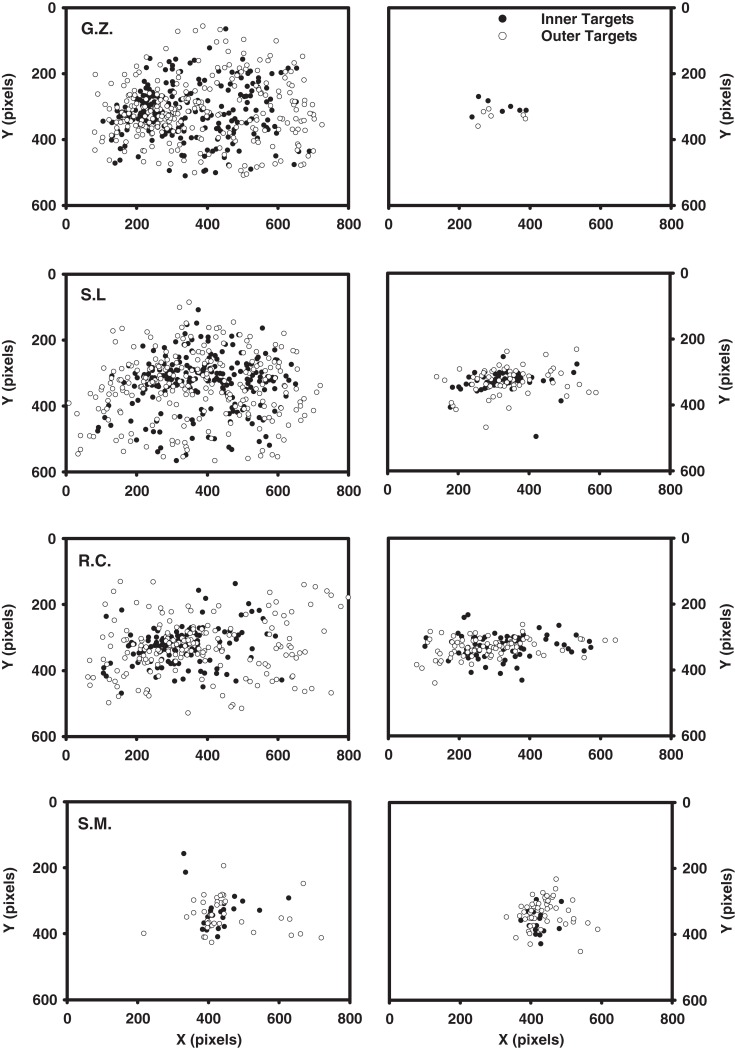
**Scatterplots of fixations for the Experiment 1 observers, excluding initial and final fixations**. Each plot includes the data from all correct target-present trials for both display sizes and target eccentricities. Static condition fixations are shown on the left; dynamic condition fixations are shown on the right.

Table 3 shows the mean number of fixations for each observer and Table 4 shows the mean fixation durations for single-fixation trials and the first, second, and final fixation durations for multiple fixation trials. Figure 3 shows scatterplots of fixations for individual observers on correct target-present trials^3^

**Table 3:** **Mean number of fixations for static and dynamic search**.

	Target-present								Target-absent			
Condition	Static				Dynamic				Static		Dynamic	
Set size	9		17		9		17		9	17	9	17
Eccentricity	Inner	Outer	Inner	Outer	Inner	Outer	Inner	Outer				
G.Z.	4.97	5.43	5.46	6.43	1.81	1.58	1.69	1.71	8.30	9.55	1.70	1.82
S.L.	4.50	6.07	5.68	7.50	1.97	2.86	3.03	3.30	7.40	9.38	3.38	3.95
R.C.	3.44	4.19	3.73	5.21	2.60	3.00	3.00	3.29	5.33	7.51	3.95	3.84
S.M.	1.26	1.62	1.92	2.32	1.28	2.34	1.69	3.47	1.96	3.08	2.69	2.98
Mean	3.54	4.33	4.20	5.37	1.91	2.44	2.35	2.94	5.75	7.38	2.93	3.15

*Data are from correct trials. Trials in which no saccades were made were included in the averages*.

**Table 4:** **Mean single, first, second, and final fixation durations for static and dynamic search (in ms)**.

	Target-present								Target-absent			
Condition	Static				Dynamic				Static		Dynamic	
Set size	9		17		9		17		9	17	9	17
Eccentricity	Inner	Outer	Inner	Outer	Inner	Outer	Inner	Outer				
G.Z.												
Single					1098	1356	1230	1330			1940	1816
First	179	183	163	173	178	165	136	203	165	168	214	253
Second	154	139	152	154	928	1058	924	1171	122	147	1350	1223
Final	214	236	266	200	1033	1165	1108	1301	148	149	1480	1535
S.L.												
Single					1892	2333	2173	2269			2468	2554
First	215	211	233	253	398	704	699	669	214	265	1088	903
Second	175	143	133	180	752	529	561	1052	166	172	524	487
Final	313	304	316	289	1100	703	652	905	176	231	569	547
R.C.												
Single					970	1274	758	1797			1449	
First	206	205	221	210	313	366	367	313	214	227	320	323
Second	156	147	155	147	445	367	415	418	170	144	533	476
Final	258	240	293	264	397	383	479	484	177	192	562	539
S.M.												
Single	610	763	625	919	689	992	690	1003	1001	1340	1212	1310
First	454	574	479	645	515	692	439	512	606	647	623	611
Second	145	335	226	168	317	409	269	210	351	404	423	429
Final	211	308	235	328	335	467	331	285	391	504	579	542

*Data are from correct responses. Trials in which no saccades were made were excluded from the first, second, and final fixation duration averages*.

Comparing the static and dynamic fixation data for observers G.Z., S.L., and R.C. reveals two clear patterns. First, there were fewer fixations in the dynamic (*M* = 2.5) condition compared to the static (*M* = 5.2). This pattern is consistent with our suggestion that a multi-location sit-and-wait strategy was used to search dynamic displays. Second, observers in the dynamic condition failed to make any eye movements on 28% of the target-present trials and 21% of the target-absent trials, but not a single static trial was completed without an eye movement. Note that both of these data patterns are highly counterintuitive given that search times averaged 496 ms longer in the dynamic condition, thereby providing more of an opportunity for gaze shifts to have occurred. Third, as clearly shown in Figure 3, fixations in the static condition are widely dispersed and vary with target eccentricity, precisely the pattern expected if gaze were linked to a search process that was actively inspecting individual items. Fixations in the dynamic condition, however, are clustered near the display’s center and are far less influenced by target eccentricity. Not only did these observers tend to move their gaze less frequently in the dynamic condition, but when eye movements did occur, they tended not to carry gaze far from the display’s center. Such a constrained pattern of fixations is exactly what would be expected from an observer waiting for a target to appear within a prescribed display region.

The oculomotor data patterns from observers G.Z., S.L., and R.C. were most consistent in the static search condition. For each observer, the number of fixations accompanying static search (but not fixation duration) increased as a function of both set size and target eccentricity. In fact, fixation duration remained relatively constant across eye movements (187 ms), with first fixations being only slightly longer than the average duration of all the other fixations (204 vs. 177 ms). Consistent with our suggestion of an active acquisition strategy underlying static search, these observers therefore responded to the increased processing demands associated with the set size and eccentricity manipulations by making more eye movements, not by increasing the duration of individual fixations.

While remaining relatively consistent amongst themselves, observers G.Z., S.L., and R.C. exhibited very different oculomotor patterns when searching dynamic displays. In contrast to their static search behavior, the number of fixations in the dynamic condition did not vary appreciably with either set size or target eccentricity. Rather, the increased processing load expected from these manipulations was expressed as longer fixation durations. Consistent with the use of a sit-and-wait strategy, their average fixation duration in the dynamic condition was 677 ms, 490 ms longer than their average fixation duration in the static condition. Unfortunately, further specifying the variation in fixation duration is complicated by the fact that multiple eye movements were often made even in the dynamic search condition. Fixations in a search trial likely serve different functions, so it is important not to treat all of these dynamic fixations as being equivalent. Some are probably no more than brief stopover points as an observer settles on a display location before actually conducting the search. For these fixations one would expect little or no effect of the independent variables on duration. Of course other dynamic fixations will more directly reflect search processing, and for these fixations one would expect an effect of the search manipulations. To obtain a clearer picture of any strategy engaged during dynamic search, we therefore attempt a brief fixation-by-fixation description of the oculomotor behavior from individual observers.

Turning first to G.Z.’s dynamic condition data, we see no systematic influence of either set size or target eccentricity on the first-fixation durations. Note from Table 4 however that these fixations were quite brief, suggesting that this observer had adopted a strategy of immediately shifting gaze upon the dynamic search display’s onset. Looking at the scatterplot of G.Z.’s fixations, this strategy apparently was to make a small leftward gaze shift, when this observer shifted gaze at all (40% of his correct responses were made without an eye movement). This brief initial fixation was followed by a much longer second fixation, with its duration tending to increase as a function of both set size and target eccentricity.

Like observer G.Z., S.L. often searched the dynamic displays without making an eye movement. Again consistent with a sit-and-wait strategy, when this observer did not shift gaze, clear effects of set size and target eccentricity emerged in her single-fixation durations. Also like G.Z., this observer made leftward gaze shifts on those trials in which she did move her eyes. These eye movements, however, were more pronounced than G.Z.’s and often resulted in gaze being repositioned quite far from the display’s center. This more extensive use of eye movements complicates an interpretation of the resulting fixation durations. When the display contained only nine items, S.L. used her initial fixation to search for the dynamic target, as indicated by the large effect of eccentricity in the nine-item trials (306 ms). However, when the display was more cluttered (i.e., the 17-item trials), S.L. shifted the bulk of her search processing to the second fixations, resulting again in a sizeable eccentricity effect (491 ms). Regardless of the fixation during which S.L. chose to conduct her search, clear differences again emerged between the static and dynamic conditions. S.L. freely used eye movements to search the static displays, with the durations of these fixations being brief and unrelated to the search manipulations. Fixations were far less frequent in S.L.’s dynamic search data, with the duration of at least one of these fixations showing evidence for an eccentricity or set size effect. For these two observers, we see the very clear emergence of two distinct patterns in the eye data, one indicating the use of a sit-and-wait strategy to search dynamic displays and the other indicating the use of an active acquisition strategy to search static displays.

Observer R.C.’s search of the dynamic displays deviated from the signature sit-and-wait patterns found for G.Z. and S.L. in two respects. First, he moved his gaze more frequently in the dynamic condition, making an average of 2 saccades compared to the 1.2 saccade average from G.Z. and S.L. Second, his fixation durations were shorter than those of the other observers, and these durations showed no clear effects of the search manipulations. Only in the final fixation durations of multi-saccade trials was there a suggestion of a set size effect. These patterns do not mean, however, that R.C. was applying the same search strategy to both the static and dynamic tasks. As can be seen from his scatterplot data, eye movements in the dynamic search condition were still far less frequent and more spatially constrained relative to those in the static condition. Also notice the pronounced tendency to make directly leftward eye movements relative to the display’s midpoint, similar to the pattern observed in S.L.’s eye data. These leftward eye movements, rather than being an attempt to fixate individual display items, appear instead to be a strategic attempt to position gaze at a region in the display deemed favorable to dynamic search. Upon closer analysis of these fixation locations, we found that R.C. was far more likely than the other observers to position gaze halfway between the two target eccentricities. This strategic allocation of gaze suggests that R.C. may have learned, either implicitly or explicitly, where targets were most likely to appear in the display. It might also explain why R.C., unlike G.Z. and S.L., showed no eccentricity effect in his dynamic condition fixation durations or RT × Set Size slopes (25 ms/item for the inner-eccentricity targets; 23 ms/item for the outer-eccentricity targets), as well as the fact that this observer made the fewest errors in the dynamic task. We return to this point in the next section.

Our final observer, S.M., showed no obvious differences in oculomotor behavior between the static and dynamic search tasks. The number of fixations made in the two search conditions did not meaningfully differ, nor were there compelling differences in the spatial dispersal of these fixations. In both tasks, S.M. made relatively few saccades that failed to carry gaze far from the display’s center – patterns that we have been interpreting as evidence for a sit-and-wait search strategy. The reason for this contradictory pattern was made clear during debriefing when S.M. volunteered the fact that he had consciously attempted to perform both the static and dynamic tasks without moving gaze. Returning to Figure 3, it appears that S.M. was largely successful in achieving this goal, although he clearly could not exert perfect control over his oculomotor behavior.

This self-imposed “don’t move your eyes” instruction provides a unique opportunity to compare static and dynamic search behavior in the near absence of eye movement. It is important, however, not to confuse a “don’t move your eyes” search strategy with a sit-and-wait search strategy. Although a sit-and-wait strategy does predict fewer fixations during search, recall that its defining characteristic is a restriction of processing to a region of the search display surrounding fixation. When both of these criteria are considered, we find that S.M.’s dynamic search performance is well described by such a strategy. Like G.Z. and S.L., the other observers who were consistently sitting and waiting for the dynamic target, S.M. was able to successfully search the dynamic displays only when targets appeared at the inner-eccentricity. Indeed, S.M.’s accuracy in the 17-item outer-eccentricity condition was at chance. We interpret this pattern as suggesting that outer-eccentricity targets often went undetected in the dynamic condition because they fell outside the display region monitored as part of the sit-and-wait strategy. If S.M. was using a sit-and-wait strategy to search the static displays, we would therefore expect a similar pattern of misses. However, as indicated in Table 2, S.M. was able to perform the static search task with a high degree of accuracy regardless of target eccentricity. We must therefore conclude that S.M. was not using a sit-and-wait strategy in the static search condition, but was instead engaging covert processes to actively search for the target.

To summarize, the eye data from Experiment 1 revealed distinct oculomotor patterns in the static and dynamic search conditions. With the exception of S.M., observers relied on eye movements to search static displays, with the number and dispersion of these fixations varying as a function of set size and target eccentricity. The number of fixations increased with display set size, and fixations were fewer and more spatially constrained when targets appeared at the inner-eccentricity relative to the outer-eccentricity. Fixation durations also remained fairly constant across these manipulations, suggesting the inspection of a fixed number of items during each fixation. All of these patterns are consistent with what we are calling an active acquisition search strategy. Observers displayed a very different search pattern when searching dynamic displays. Eye movements were relatively infrequent and, when they did occur, failed to move gaze far from the display’s center. The number of fixations during dynamic search also failed to show a clear effect of set size and target eccentricity. Fixation durations, however, were in general much longer than their static condition counterparts and increased as a function of these two manipulations, albeit idiosyncratically both within and across observers. These patterns are consistent with what we are calling a multi-location sit-and-wait search strategy.

Given the existence of these two search strategies, the question remains as to *why* observers would choose to use one strategy in the static condition and the other in the dynamic condition? Answering this question is straightforward in the case of static search; because items are not jumping from location to location in these displays, sitting and waiting for a target to appear would be foolhardy, making an active acquisition search strategy the only alternative. More interesting is to consider why observers chose to adopt a sit-and-wait strategy for their dynamic search. Unlike the case of static search, observers searching dynamic displays were highly motivated to avoid eye movement and to adopt a sit-and-wait strategy. This motivation likely arose from several sources. First, “covering” a dynamic display with one’s high-resolution fovea would no longer guarantee the target’s central inspection. From the observers’ perspective, the small cost of making eye movements would therefore not be offset by any coverage benefit. A second factor motivating a sit-and-wait search is that the dynamic display items were simply moving too fast to provide a good target for a saccade. Whatever the reasons for the different oculomotor behavior during static and dynamic search, it is clear that the stimuli in these two search tasks would be differentially perceived and processed, and that Horowitz and Wolfe (1998) should have considered these differences before concluding that a single process underlies these search conditions.

The general patterns of oculomotor behavior observed in Experiment 1 also correspond to those reported by Geyer et al. (2007). In their experiment, fixation number, but not fixation duration, increased with set size for static search displays, whereas the opposite pattern was found for dynamic search. Although target eccentricity did vary in their experiment, it was not manipulated systematically, nor were the data analyzed with respect to target eccentricity, so it is unknown how this variable affected any of the oculomotor variables during search of either static or dynamic displays. In addition, they presented oculomotor data averaged across observers. Our individual observer analyses suggest that there may be a high degree of variability in how different individuals implement a relatively passive sit-and-wait strategy when searching dynamic displays. Thus, our findings extend current knowledge of oculomotor behavior during search of dynamic displays by elucidating effects of target eccentricity as well as illustrating the degree of variability that isn’t captured by comparisons of means of oculomotor variables.

#### Modeling a multi-location sit-and-wait search strategy

From Experiment 1 we know that observers in the dynamic search condition often constrained their fixations to the display’s center. We also discussed the consequences of this behavior for search and some of the reasons why observers may have adopted such a sit-and-wait search strategy. What remains to be discussed is an explanation of how this strategy relates to gaze position and the deployment of attention in a dynamic search task, and it is to these questions that we now turn.

We propose a simple relationship between gaze position and the deployment of attention in dynamic displays. Following Wolfe et al. (1998), we suggest that the deployment of attention begins at the current fixation location and proceeds outward. However, because of the loss of search history accompanying each new dynamic frame, we propose that observers halt this outward progression in the dynamic condition and reset their attention back to fixation following each display change. This strategic resetting of attention to fixation would in turn prevent the inspection of more eccentric display locations, resulting in detection failures increasing with target eccentricity. In the case of the static task, however, once items near fixation had been inspected, attention could be deployed to increasingly eccentric items until processing eventually reaches the target. It is this opportunity for thorough display inspection that we believe results in lower error rates in the static search condition.

The above sketch of our sit-and-wait model suggests that accuracy of target detection in the dynamic search condition should be limited by two interacting factors. First, search accuracy should improve with the number of locations that can be monitored during a dynamic trial (sample size). Second, dynamic search performance will depend on the distance between the target’s location in the search display and the observer’s locus of gaze. Given a central attentional bias (Wolfe et al., 1998), and the use of a resetting operation during dynamic search, observers electing to maintain gaze at the display’s center might have inadequate opportunity for processing to extend to more eccentric display locations during a single dynamic frame. Such a spatiotemporal processing constraint, in addition to restricting attention to the same set of display locations (i.e., the sample set), would also result in misses increasing with target eccentricity. If observers were not constrained by this resetting operation, then the outward deployment of attention would instead continue where it left off on the previous frame and no differential effects of eccentricity on errors would be expected between static and dynamic search.

**Figure 4:**
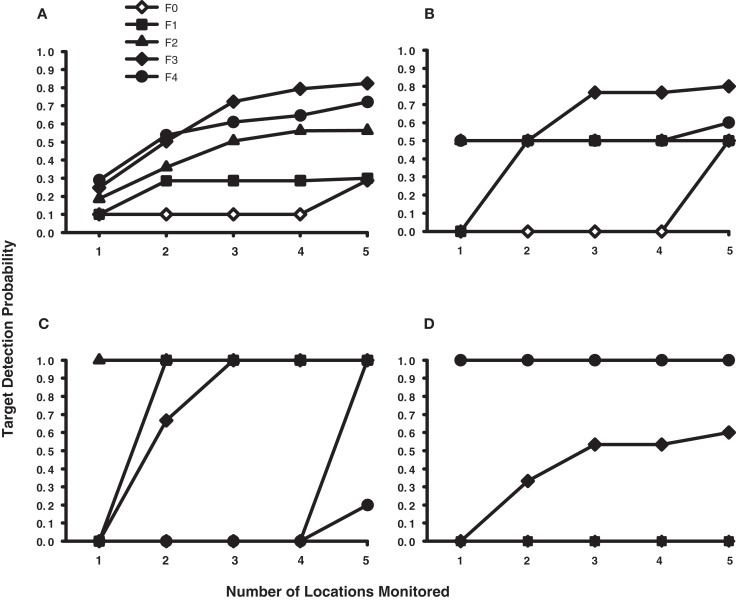
**Simulated probability of target detection for a sit-and-wait strategy as a function of gaze position in the display and the number of locations monitored during the dynamic trial**. **(A)** Probability functions for the dynamic task used by Horowitz and Wolfe (1998). **(B)** Probability functions for the dynamic task used in the current study, collapsed across eccentricity. **(C)** Simulation data for dynamic targets appearing at the inner eccentricity. **(D)** Simulation data for dynamic targets appearing at the outer eccentricity. The F0 function indicates the probability of target detection when gaze is positioned at the center of the display; the F1–F4 functions indicate the probability of target detection when gaze is positioned at the first, second, third, and fourth item eccentricities. See the Appendix for details.

These sample size and eccentricity constraints on dynamic search can be formally modeled within the context of a multi-location sit-and-wait strategy. The success of a sit-and-wait strategy depends on the probability of the target appearing among the set of monitored display locations, which in turn depends on the size of this set, the position of gaze in the display, and ultimately on the display contingencies governing target location in the dynamic search task. If one knows these contingencies, it is therefore possible to plot the probabilities of successful sit-and-wait target detection as a function of sample size (von Mühlenen et al., 2003) and gaze position. Figure 4A shows five of these Detection Probability × Sample Size functions for the dynamic search task used in Horowitz and Wolfe (1998). Each function (F0–F4) corresponds to a different fixation position in the displays where a hypothetical observer would have elected to sit-and-wait for the dynamic target. These five locations were chosen such that each describes an increasing eccentricity relative to initial fixation, with the F0 function describing a sit-and-wait locus at the display’s center (0° eccentricity) and the F1–F4 functions describing sit-and-wait locations at 2°, 4°, 6°, and 8°, respectively. The equations used to derive these probability functions are provided in the Appendix, along with additional procedural details. In all cases, attention was assumed to be reset with the onset of each dynamic frame, thus restricting the locations monitored to those nearest fixation.

From the figure it is clear that the probability of correctly detecting a target in Horowitz and Wolfe’s (1998) dynamic search condition would increase with the number of locations monitored and the distance that gaze shifted from the display’s center. If gaze were positioned at or near the display’s center (functions F0 and F1), the probability of detecting a target would be quite low across a wide range of sampled locations. However, if observers had learned to make a 6–8° eye movement following a dynamic trial’s onset (functions F3 and F4), and then monitored at least five locations while sitting at this new fixation position, their detection probability would average 0.77 – putting the expected miss rate near the levels reported in Horowitz and Wolfe (1998). As already noted, Horowitz and Wolfe (1998) may have unintentionally motivated their observers to adopt such an oculomotor response by having targets in the dynamic displays appear disproportionately in peripheral visual locations. Based on these modeling results, it therefore seems that Horowitz and Wolfe (1998), while justified in dismissing a single-location sit-and-wait search strategy as an explanation for their dynamic condition data, are not justified in extending their probabilistic argument to a multi-location sit-and-wait strategy (see also von Mühlenen et al., 2003), particularly when observers were free to reposition their gaze over the displays.

Figure 4B through Figure 4D show similar probability functions plotted for the dynamic search task used in Experiment 1. These results are different from our analysis of the Horowitz and Wolfe (1998) data because of the different display contingencies used in the two studies. Recall that targets in the Horowitz and Wolfe (1998) study appeared at every display location with equal probability whereas targets in our task appeared randomly and with equal probability at only the second (3.1°) or fourth (6.2°) display eccentricities. To better illustrate the effect of eccentricity on the success of a sit-and-wait search strategy, we show the simulation data both combined across target eccentricity (Figure 4B) as well as separately for inner-eccentricity (Figure 4C) and outer-eccentricity (Figure 4D) dynamic conditions. Comparing Figures 4A and 4B illustrates how even seemingly minor differences in target display contingencies can have a dramatic impact on the expected performance of a sit-and-wait search strategy. Although both figures are similar in that expected detection probability tends to increase with sample size, we see in Figure 4B that this increase is now more closely tied to where the targets appeared in the dynamic displays and the distance that gaze moved from fixation. Monitoring up to five locations yields a chance probability of target detection when gaze is positioned at the display’s center and the first and second item eccentricities (F0–F2, respectively), and is only slightly better than chance when gaze is located at the fourth item eccentricity (F4). However, when gaze is located at the third item eccentricity (F3), expected probability of detection increases sharply to 0.8.

Segregating the simulation data by eccentricity helps to clarify the Figure 4B patterns. When targets appear at the inner-eccentricity (Figure 4C), and up to four locations are monitored over dynamic frames, a sit-and-wait locus at the display’s center yields an expected detection probability of zero because only the four 1.5° locations nearest fixation would be processed. However, if five locations can be monitored, one location from the 3.1° inner target ring would now be included within the monitored set, resulting in the probability of detection jumping immediately to 1.0. Contrast this scenario with the detection probabilities expected in the outer-eccentricity condition (Figure 4D). Again assuming fixation at the display’s center, we now find that the probability of target detection is zero even when five (and many more) locations are monitored. The reason for this detection failure is because 29 locations would require monitoring before processing would extend to the outer target ring – an unlikely event given the current stimuli.

Of course higher detection probabilities would be expected as gaze moves closer to the target rings in their respective eccentricity conditions. For example, in the inner-eccentricity condition, only a single-location need be monitored to achieve a 1.0 detection probability when gaze is positioned on the 3.1° target ring (F2). When gaze is positioned at the first (1.5°) eccentricity and two locations are monitored, the nearest-to-fixation constraint requires that one of these locations be on the inner target ring, again resulting in a 1.0 detection probability (F1). For inner-eccentricity targets, the least successful display locations in which to apply a sit-and-wait search strategy are at the 6.2° target ring (F4) and at the display’s center (F0). Predictably, these probabilities differ in the case of outer-eccentricity targets, with high detection probabilities expected only for fixations on the 6.2° target ring (F4). Fixations on the 4.6° locations (F3) should yield an intermediate level of detection, whereas sitting and waiting at the first two eccentricities and at the display’s center should not produce above-chance target detection for the sample sizes used in our simulations.

Returning to Table 2 and the dynamic search performance of our individual observers, we see that many of the above patterns predicted by our multi-location sit-and-wait model are reflected in the behavioral data. Consistent with the model, observers who maintained gaze at the display’s center (S.M., S.L., and G.Z.) frequently missed outer-eccentricity targets. In fact, the near chance performance of these observers in the 17-item outer-eccentricity condition is almost perfectly described by the F0–F2 functions in Figure 4D, which show a zero probability of target detection. Because these simulations used a fixation point at or near the display’s center, targets located at eccentric locations would rarely be detected, thereby requiring observers to frequently guess when making a response. Our sit-and-wait model also nicely describes the dynamic search performance of observer R.C., despite his dramatically different oculomotor behavior. Recall that R.C. often shifted his gaze in the dynamic task quite far from center (Figure 3), and, perhaps as a result of this oculomotor search strategy, made the fewest misses from among our four observers. As illustrated in Figure 4B, our model predicts the identical relationship between target detection and gaze position. When more than two locations are monitored during this dynamic task, positioning gaze midway between the two target eccentricities (F3) yields a higher expected probability of target detection than when gaze is positioned directly on either the inner or outer target ring.

A multi-location sit-and-wait strategy therefore provides a good account of the Experiment 1 data, both in terms of the high dynamic condition miss rates when targets appeared at the outer eccentricity and observers elected to keep gaze near the display’s center, as well as the improved accuracy for our one observer who chose to shift his gaze to more eccentric display locations. Whether this observer had learned that targets were more likely to appear at these eccentric locations and was deliberately shifting gaze, or was simply more prone to oculomotor activity and therefore more likely to position gaze nearer the targets, we cannot say. Nor can we say with certainty whether Horowitz and Wolfe’s (1998) observers, or those of von Mühlenen et al. (2003), or of Geyer et al. (2007) were biased by the target contingencies into adopting a search strategy similar to the one demonstrated by R.C. However, we do know that if their observers learned, whether explicitly or implicitly, of these target contingencies, they too might have adopted more favorable display locations in which to apply a multi-location sit-and-wait strategy, thereby avoiding the high dynamic condition miss rates reported in the current investigation.

## Experiment 2

The previous section showed that a multi-location sit-and-wait strategy, when modulated by gaze position, offers a reasonable description of the misses generated by our Experiment 1 observers performing a dynamic search task. However, this demonstration was *post hoc* and limited by the observers’ success in finding good display locations in which to sit and wait for the target. As illustrated in Figure 4, some display locations are far more optimal than others, and even our best observer, R.C., managed to find these optimal locations in only 22% of the trials.

In Experiment 2 we seek to examine more systematically this relationship between gaze position and accuracy in a dynamic search task by requiring observers to maintain fixation either at the display’s center or at a point midway between the two target eccentricities, which should be an optimal location to sit and wait for the target (Figure 4). This manipulation provides a critical test of the multi-location sit-and-wait strategy. If observers use this strategy to search dynamic displays, their accuracy should be higher when gaze is positioned at the eccentric location compared to when gaze is at the display’s center. However, if this optimal sit-and-wait positioning fails to produce an accuracy benefit, then we can reject the hypothesis that observers were using this strategy to conduct their search. A secondary prediction relating fixation position to expected eccentricity effects also follows from this manipulation. Positioning gaze midway between the two target eccentricities should yield a smaller eccentricity effect compared to when gaze is held at the display’s center and the targets actually appear at different visual eccentricities from trial to trial. Note however that this relationship is complicated by the fact that display items will be asymmetrically distributed around fixation when gaze is positioned at an eccentric location, so we consider this prediction to be more tenuous than the first.

### Materials and methods

#### Participants

Twenty four students recruited from the University of Delaware, who were each paid $10/h and had normal or corrected-to-normal vision, participated in this experiment.

#### Apparatus and stimuli

Eye movement and manual data were collected using the EyeLink II video-based eye tracking system (SR Research, Ltd.). Eye position was sampled at a rate of 500 Hz, the system’s spatial resolution was estimated to be 0.2°, and changes in gaze position were available to the computer running the display program within 8 ms. All displays were presented on a 21″ Dell SVGA monitor with a refresh rate of 100 Hz. Search displays were presented at a screen resolution of 800 × 600 pixels. Observers’ head position and viewing distance were fixed with a chinrest, and all responses were made with a GamePad controller attached to the computer’s USB port. Search judgments were made with the left and right index-finger buttons; trials were initiated with a button operated by the right thumb. The stimulus and display characteristics were the same as in the previous experiment. We attached a white rectangular cardboard frame to the monitor’s screen so the search displays would fill the screen to the same degree as in Experiment 1.

#### Procedure and design

The methodology was identical to that of Experiment 1 with the following exceptions. First, there were now two dynamic display conditions and no static search condition. As in Experiment 1, the *central* condition used a fixation cross to position the observer’s gaze at the dynamic display’s center. In the *eccentric* condition, the fixation cross was presented at an eccentricity of 4.6°, midway between the 3.1° and 6.2° rings where a target would appear in a target-present trial. Second, we removed the RT feedback following each trial and presented only accuracy feedback. We did this out of concern that RT information might have biased observers to make overly fast responses, thereby contributing to the high miss rates reported in Experiment 1. Third, observers were instructed to maintain fixation on the central or eccentric cross, which remained visible throughout each trial. Observers were also given fixation feedback at the end of each trial in the same location as the fixation cross (if fixation was maintained, the accuracy feedback was shown in green font; if an eye movement occurred, the accuracy feedback was shown in red font).

Fixation position was a between-subjects manipulation, with the central and eccentric groups each having 12 randomly assigned observers. In the eccentric group, half of the observers were shown a fixation cross on the left side of the screen; the other half were shown a fixation cross in the corresponding location on the right side of the screen. Within each fixation condition, trials were divided evenly between two target conditions (present vs. absent), two set sizes (9 or 17 items), and two target eccentricities (3.1° or 6.2° from the display’s center, which we will refer to as *inner* and *outer*, respectively), yielding 40 trials per target-present cell and 80 trials per target-absent cell. Observers were given one block of 32 practice trials followed by four blocks of 80 trials, for a total of 320 experimental trials.

### Results

Trials in which the observer’s gaze shifted more than 1° from the fixation cross (3.3%) were not included in any analyses. Before evaluating either of our hypotheses, we converted the miss and false alarm rates for the two conditions to *d*′ values. We did this because the difference in false alarm rates in the two conditions, shown in Table 1, indicates that a direct comparison of miss rates would not be appropriate. Overall, observers made more false alarms in the central fixation condition [21.7 vs. 10.3%, *F*(1, 22) = 12.2, *p* < 0.05]. Although the model’s predictions are expressed in terms of miss rates, the pattern of misses generated by the model would correspond directly to *d*′ values. This is because the model can be considered perfectly conservative in its response bias. As discussed previously, objects selected by the model were represented discretely (not probabilistically or noisily) as either the target or a distractor. The model made a “target-present” response only when the target was one of the selected objects; thus, it was incapable of producing false alarms.

**Figure 5:**
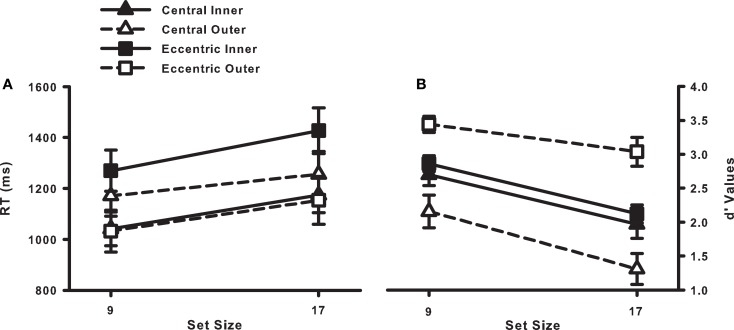
**(A)** RT × Set Size functions for correct target-present responses from Experiment 2. **(B)** *d*′ Values as a function of set size, fixation position (central or eccentric), and eccentricity (inner or outer). Error bars indicate the standard error of the mean.

Our critical hypothesis, that search should be more accurate with an eccentric fixation position than a central fixation position, was supported by the *d*′ values, shown in Figure 5B. Overall, *d*′ values were higher for the eccentric fixation condition [2.03 vs. 2.87, central vs. eccentric; *F*(1, 22) = 15.9, *p* < 0.05]. What’s more, the RT data (Figure 5A) suggest that this was not the result of a speed–accuracy tradeoff. Neither target-present RTs [1160 vs. 1221 ms, central vs. eccentric; *F* < 1] nor target-absent RTs [1651 vs. 1951 ms, central vs. eccentric; *F*(1, 22) = 3.03, *p* > 0.05] differed significantly between the two fixation conditions. Our prediction of improved accuracy in the eccentric fixation condition was therefore upheld, providing empirical and converging evidence supporting the use of a multi-location sit-and-wait strategy in this dynamic search task.

Our secondary hypothesis, that eccentricity effects should be attenuated in the eccentric fixation condition relative to the central fixation condition, was also supported by the *d*′ data, although in a somewhat unexpected direction, as shown in Figure 5B. The fixation condition × target eccentricity interaction was significant, *F*(1, 22) = 34.55, *p* < 0.05. For the central fixation condition, *d*′ values were significantly lower for outer-eccentricity targets than for inner-eccentricity targets, as predicted [1.73 vs. 2.33, outer vs. inner; *t*(11) = 3.05, *p* < 0.05]. For the eccentric fixation condition, however, *d*′ values were significantly higher for outer-eccentricity targets than for inner-eccentricity targets [3.24 vs. 2.49, outer vs. inner; *t*(11) = 6.37, *p* < 0.05].

Observers who fixated midway between the two target eccentricities were able to respond more accurately to targets at the outer-eccentricity relative to the inner-eccentricity. This reverse eccentricity effect was not the by-product of a speed-accuracy tradeoff as an analysis of RTs revealed the same pattern observed in the *d*′ data: RTs decreased with target eccentricity in the eccentric fixation data [1348 vs. 1093 ms, *F*(1, 11) = 25.3, *p* < 0.05], but they increased in the central fixation data [1107 vs. 1213 ms, *F*(1, 11) = 12.6, *p* < 0.05]. It thus appears that fixation position and target eccentricity did interact, but this interaction was not in the direction that we predicted.

At this time, we can only speculate as to the cause of the observed eccentricity effects in the eccentric fixation condition. One possibility is that targets appearing in the outer display ring were more salient under fixed-gaze conditions compared to targets appearing in the inner-ring, perhaps due to differential crowding effects (inner-ring targets are flanked on both sides by distractors, outer-ring targets are flanked on only one side). This differential crowding, combined with the broad distribution of attention likely resulting from observers searching without moving gaze, might have produced an accuracy advantage for outer-eccentricity targets in the eccentric fixation condition. More work will be needed to substantiate this hypothetical relationship between target eccentricity, gaze position, viewing condition (free vs. fixed), and accuracy in a dynamic search task.

## General Discussion

Many of our day-to-day search tasks take place in a dynamically changing environment. Whether we are searching for a fly that has invaded our home, or are looking for a fish in an aquarium or a favorite duck in a pond, we are searching for a target that could be almost anywhere at any given moment. The current study advanced understanding of this neglected variety of search task by first documenting the oculomotor behavior accompanying dynamic search and comparing it to static search performance, then by developing and testing a model of dynamic search.

Initially, the dynamic search paradigm was used by Horowitz and Wolfe (1998) to challenge the assumption of distractor memory use during search – it was simply a comparison condition in which distractor memory could not be used. Central to the logic of their study was the premise that the static and dynamic search tasks tap into the same underlying search process, and that the information used by static search can be directly inferred from dynamic search performance. The present study suggests two reasons to question this premise. First, although the manual data from Experiment 1 yielded patterns qualitatively similar to the key findings from Horowitz and Wolfe (1998), we also observed in these data a pronounced eccentricity effect that differentially affected miss rates in the static and dynamic search conditions. Based on this finding, we hypothesize that display contingencies, notably the higher probability of a target appearing in peripheral display locations relative to more central locations, might have influenced dynamic search efficiency in previous experiments (Horowitz and Wolfe, 1998; von Mühlenen et al., 2003; Geyer et al., 2007; Wang et al., 2010). Second, we found dramatic differences in the oculomotor behavior of observers participating in the dynamic and static search tasks. Most notably, fixations in the dynamic task were largely constrained to the central portion of the display whereas fixations in the static task were distributed far more uniformly over the display items. These oculomotor differences suggest that attention may have been allocated differently in the two search conditions, again making any assumption of a common process problematic.

To better understand the processes enlisted during dynamic search, we conducted an individual observer analysis of the eye movement data from Experiment 1. One strikingly clear finding from this analysis was that there is considerable individual variability in how one might conduct a dynamic search. In even our small sample of four observers, only G.Z. and S.M. adopted a similar dynamic search strategy, one that involved holding gaze near the display’s center. Our third observer, R.C., distributed his fixations far more broadly over the search display, and our fourth observer, S.L., seemed to adopt a hybrid strategy of lingering at the display’s center and then making a large-amplitude saccade relatively late in the dynamic search trial. A second and equally clear finding from this analysis was that although observers can, and do, adopt different dynamic search strategies, not all of these strategies are equally effective. Of our four observers, only R.C. was successful in keeping his error rates low regardless of target eccentricity. Given their wildly varying error rates, and the fact that some observers chose to move their eyes whereas other did not, it is misleading to ignore these individual strategic differences by collapsing performance into an “average” measure of dynamic search behavior, as Geyer et al. (2007) chose to do.

Although such individual differences complicate any simple theoretical account of dynamic search performance, we believe that much of our data, and the data from Horowitz and Wolfe (1998), might be explained by a multi-location sit-and-wait strategy. Consistent with a proposal by von Mühlenen et al. (2003), we developed a formal sit-and-wait search model that monitors a variable number of display locations surrounding fixation and resets itself to this fixation point following the onset of each new dynamic frame. Using this formal model, we demonstrated that even a slight relaxation of the strict single-location processing constraint assumed by Horowitz and Wolfe (1998) can enable a sit-and-wait strategy to describe the successes and failures of our Experiment 1 observers in detecting dynamic targets. Those observers who elected to park gaze near the display’s center and wait for the target to appear at one of the inner-eccentricity positions predictably missed targets when they appeared at the outer eccentricity. The region of positions being monitored around fixation apparently did not extend to these more distant display locations. However, observers less reluctant to shift gaze under dynamic viewing conditions were more likely to find display positions better suited to a sit-and-wait search, and were consequently able to perform the dynamic task with a higher degree of accuracy.

In Experiment 2 we manipulated gaze position in order to test our proposed relationship between eye position and the locus of a sit-and-wait strategy, as well as our suggestion that some sit-and-wait display locations are better than others because of target-presentation contingencies. As predicted by our fixation-modulated sit-and-wait model, we found that positioning gaze midway between two target eccentricities resulted in improved accuracy relative to performance when gaze was positioned at the display’s center. We will continue to explore this effect of gaze position on dynamic search with a future version of our model, one that allows the sit-and-wait locus to be updated during a trial. We plan to use the gaze positions from individual observers to dynamically position a sit-and-wait process as it actually existed on each fixation of every dynamic trial, thereby enabling more realistic fits between our sit-and-wait model and human search behavior under free viewing conditions.

## Conflict of Interest Statement

The authors declare that the research was conducted in the absence of any commercial or financial relationships that could be construed as a potential conflict of interest.

## Footnotes

^1^To ensure that no artifact of display presentation was contributing to static and dynamic search differences, a static trial consisted of 15 identical video frames drawn to the screen.

^2^We believe one factor contributing to this uncommonly shallow slope might have been the time-terminated displays in our paradigm. As discussed more fully elsewhere (Klein and MacInnes, 1999; Horowitz and Wolfe, 2001; von Mühlenen et al., 2003), an observer’s inability to exhaustively inspect dynamic display items would likely result in the adoption of a time-based rule to terminate search (Chun and Wolfe, 1996). Given that observers in our task would soon learn that the mask’s onset after 2,025 ms would disrupt search, they would likely adopt a termination criterion that anticipated mask onset. Indeed, average target-absent responses fell just short of the 2,025 ms presentation duration, supporting this hypothesis. The application of this rule would have two important consequences for search. First, it would serve to flatten the set size function and increase false alarms, the exact patterns observed in the dynamic data. Second, it would make it difficult to directly compare the target-absent and the target-present data because each would be using a different response criterion. Given that our goal was to study search and not the selection of termination rules under dynamic viewing conditions, we focus the remainder of our discussion on the target-present data.

## Appendix

Horowitz and Wolfe’s (1998) argument against a single-location sit-and-wait strategy was formalized as:

(A1) $$p\left(\text{hit}\right)=\overset{4}{\underset{i=1}{\sum }}p\left(E_{i}\right)p\left(S_{i}\right)p\left(T_{i}|E_{i}\right),$$

where *p*(*E_i_*) is the probability of the target appearing at a given eccentricity, *p*(*S_i_*) the probability of a particular location being selected at that eccentricity, and *p*(*T_i_*|*E_i_*) the conditional probability that the target would appear at that location given that it appears at that eccentricity, summed over the four eccentricities. Using this formula, they determined that the probability of a target in their dynamic displays appearing at the innermost eccentricity (2°) was 4/40, the probability that an observer would select a location at that eccentricity was 4/40, and the probability that the target would appear at a given location from this eccentricity at least once in the 20 dynamic frames was 1 − (3/4)^20^, thereby yielding a 0.01 probability of selecting a location at the eccentricity where a target would appear. The corresponding probabilities for the three other target eccentricities (4°, 6°, and 8°) were 0.037, 0.074, and 0.116, respectively, resulting in a 0.237 overall probability of target detection. Given that the observed hit rate in their Experiment 2 was approximately 95%, Horowitz and Wolfe (1998) rejected a single-location sit-and-wait strategy as being a plausible explanation for dynamic search performance.

To explore the implications of relaxing this single-location constraint on a sit-and-wait strategy, we simulated 9,120 dynamic trials using the display constraints outlined in Horowitz and Wolfe (1998). For each simulated trial, 20 target locations were generated, one for each frame in a dynamic trial. These 20 locations were obtained by randomly sampling with replacement the set of display locations at the eccentricity used on that trial. In the simulation, 10% of the targets appeared at the first eccentricity, 20% at the second, 30% at the third, and 40% at the fourth, replicating the target-presentation contingencies used in the Horowitz and Wolfe (1998) study.

In modeling the use of a multi-location sit-and-wait strategy during dynamic search, we assumed that gaze could be located either at the display center or any of the 40 display locations used by Horowitz and Wolfe (1998). To determine the locations monitored by the sit-and-wait strategy, for each gaze position we calculated the four nearest display locations. Given a gaze position (*g*) and sample size (*n*), a target was labeled as “detected” if it appeared at the gaze position or the *n − 1* nearest locations to *g*. For example, if the sample size was 1, the target would have to appear at the gaze position in order to be detected. Likewise, if the sample size was 3, the target would have to appear either at the gaze position or one of the two locations nearest to it to be detected. As described in the text, embedded within our nearest-to-fixation calculation is the assumption that attentional deployments are reset to the current gaze position with each new dynamic frame’s onset. The equation describing this detection event simplifies from the Horowitz and Wolfe (1998) formulation to:

(A2) $$p\left(\text{hit}\right)=\overset{n}{\underset{i=k}{\sum }}p\left(E_{i}\right)p\left(T_{i}|E_{i}\right),$$

because *p*(*S_i_*), the probability of an observer selecting a location at a given eccentricity, would now be 1. In this equation, *i*, *n*, and *k* refer to the item eccentricities that are being monitored.

To derive the target-detection probability functions shown in Figure 4A, we then averaged the detection probabilities for each of the gaze positions at a given eccentricity and plotted this average as a function of sample size. The F1 function therefore averages over the four potential gaze positions at the first eccentricity (2°), and the F2–F4 functions compute a similar average for positions at the second (4°), third (6°), and fourth (8°) eccentricities, respectively. The F0 function is based on a single gaze position at the display’s center.

Gaze position interacts with sample size to determine the actual display locations monitored by the sit-and-wait search strategy. For example, if five display locations were being monitored, then the generation of the F1 function would use all four display locations at the innermost 2° eccentricity and one location from the 4° eccentricity. However, if we again assume that five locations are monitored but consider an F4 simulated gaze position, one location would be used from the second eccentricity, two locations from the third, and two from the fourth. Target detection probabilities can therefore be calculated by adding the probabilities of a target appearing within these monitored locations for each of the tested gaze positions and sample sizes. When a sample included more than one location at the same eccentricity, the probability of the target appearing in either location given its appearance at that eccentricity [*p*(*T*_i_|*E_i_*)] was calculated as 1 – (*n*/*N*)^20^, where *n* was the number of locations sampled at that eccentricity and *N* was the total number of locations at that eccentricity.

The procedures used to apply a sit-and-wait search strategy to the current dynamic search data (Figure 4B through Figure 4D) were identical to the ones outlined above with the following three exceptions. First, the display positions used in our study differed from those used by Horowitz and Wolfe (1998). Rather than the 2°, 4°, 6°, and 8° eccentricities used in the Horowitz and Wolfe (1998) study, the simulations for our data were based on 1.5°, 3.1°, 4.6°, and 6.2° eccentricities. Second, targets in our dynamic task were constrained to appear at only two eccentricities with equal probability; targets in the Horowitz and Wolfe (1998) study appeared at all four eccentricities and the probability of target appearance increased with eccentricity. Third, each dynamic trial in our study consisted of 15 frames rather than 20. These 15 target locations were generated by randomly sampling the 10 locations at the target eccentricity without replacement twice, then combining the two sets of locations to create the dynamic trial.
